# The effect of SGLT2 inhibitor and HIF-PHI on the podocyte-specific molecules and cytoskeleton of diabetic podocytes

**DOI:** 10.1186/s12882-025-04677-0

**Published:** 2025-12-06

**Authors:** Chuanlei Li, Jack K. C. Ng, Gordon C. K. Chan, Winston W. S. Fung, Kai-Ming Chow, Cheuk-Chun Szeto

**Affiliations:** 1 Carol & Richard Yu Peritoneal Dialysis Research Centre, Department of Medicine & Therapeutics, Prince of Wales Hospital, Shatin, Hong Kong China; 2 Li Ka Shing Institute of Health Sciences (LiHS), Faculty of Medicine, The Chinese University of Hong Kong, Shatin, Hong Kong China

**Keywords:** Diabetes, Chronic kidney disease, Proteinuria, Anemia

## Abstract

**Background:**

Sodium-glucose cotransporter 2 inhibitors (SGLT2i) and hypoxia-inducible factor prolyl hydroxylase inhibitors (HIF-PHI) have pleiotropic properties that may affect glomerular podocytes. We studied the effects of SGLT2i and HIF-PHI on cultured podocytes and human diabetic kidney disease (DKD) specimens.

**Methods:**

Cultured human podocytes were treated with high glucose, Dapagliflozin, or Roxadustat. Podocyte-associated molecules levels and morphological changes were assessed. We then studied the kidney biopsy of 5 DKD patients treated with SGLT2i and 5 untreated DKD patients (control group). The distribution patterns of podocyte-associated molecules were assessed.

**Results:**

In high glucose condition, cultured podocytes had reduced mRNA expression of nephrin, podocalyxin, and synaptopodin, which was restored by treatment with Dapagliflozin, Roxadustat, or both. The corresponding intracellular protein levels were similarly reduced in high glucose and partly restored by Dapagliflozin, Roxadustat, or both. In high glucose condition, podocyte cell bodies were shrunken, and the distribution of nephrin and podocin on cell surface became granular, which were restored to the normal linear pattern when treated with Dapagliflozin, Roxadustat, or both. In high glucose condition, podocalyxin distribution at podocyte apical membrane was disorganized, while the expression of synaptopodin was reduced in the cell processes, with the punctate appearance disrupted; Dapagliflozin, but not Roxadustat, partly restored their normal distribution. In human DKD, the disorganized nephrin, podocin, podocalyxin, and synaptopodin distribution was similar to cultured podocytes, and the disrupted distribution returned to the normal linear continuous pattern with SGLT2i treatment.

**Conclusion:**

SGLT2i Dapagliflozin and HIF-PHI Roxadustat partly restore the podocyte morphology and intracellular mRNA and protein levels of podocyte-associated molecule in a diabetic milieu.

**Clinical trial number:**

Not applicable.

**Supplementary Information:**

The online version contains supplementary material available at 10.1186/s12882-025-04677-0.

## Key Learning Points

What was known

This study adds

Potential impact

• Podocyte dysfunction plays an important role in the pathogenesis of diabetic kidney disease (DKD)

• Sodium-glucose cotransporter 2 (SGLT2) inhibitors and hypoxia-inducible factor prolyl hydroxylase inhibitors (HIF-PHI) have pleiotropic properties on glomerular podocytes.

• In cultured human podocytes, SGLT2i and HIF-PHI partly restore the podocyte morphology and intracellular mRNA and protein levels of podocyte-associated molecules in a diabetic milieu.

• The same pattern of podocyte-associated molecules changes was present in kidney biopsy from DKD patients with SGLT2i treatment.

• Our study showed that SGLT2 inhibitor and HIF-PH inhibitor are protective to podocytes in vitro, but the concomitant use of these two medications does not appear to have obvious additional effects.

**Supplementary Information:**

The online version contains supplementary material available at 10.1186/s12882-025-04677-0.

## Iniontroduct

The prevalence of type 2 diabetes has risen markedly over the past few decades [1, 2]. Diabetic kidney disease (DKD), generally regarded as a microvascular complication, affects about 30–50% of patients with type 2 diabetes [3]. The pathogenesis of DKD is multifaceted, but podocytes playing a pivotal role [4]. In patients with DKD, increased albumin excretion primarily results from alterations in the glomerular filtration barrier. The integrity of podocytes is essential for maintaining a functional glomerular filtration barrier, and podocyte injury is a characteristic feature of diabetic nephropathy. A reduction in podocyte number and detachment from the glomerular basement membrane are directly correlated with the urinary albumin excretion rate (UAER) in both type 1 and type 2 diabetes [5, 6].

In additional to the traditional renin-angiotensin-aldosterone system (RAAS) blockers, sodium-glucose cotransporter 2 (SGLT2) inhibitors are efficacious in providing renal protection for both DKD and non-diabetic chronic kidney disease (CKD) [7–9]. While the primary action of SGLT2 inhibitors occurs in the proximal tubule, evidence suggests their ability to modulate podocyte dysfunction [10]. In experimental models of diabetes, SGLT2 inhibitors have been shown to reduce albuminuria, mesangial dilation, and matrix accumulation [11], which could not be readily explained by the action of SGLT2 inhibitor on the proximal tubules, but by the effects on glomerular hemodynamics, probably coupled with the inhibition of renal inflammation and oxidative stress [12].

On the other hand, hypoxia-inducible factor prolyl hydroxylase inhibitor (HIF-PHI) has been identified as an effective treatment for anemia associated with kidney disease [13, 14]. However, some studies indicate that HIF-PHI has the off-target effect of modulating energy metabolism in the kidneys during early stages of DKD [15, 16]. Notably, Hasegawa et al. [15] showed that the instability of HIF-1α and the subsequent loss of its downstream compensatory pathways are early events in DKD. HIF-1α, activated by hypoxic conditions, initiates responses that promote angiogenesis, erythropoiesis, and changes in energy metabolism [17]. In diabetic kidneys, administration of the HIF-1α stabilizer enarodustat ameliorates metabolic and transcriptional dysregulation, indicating a metabolic shift towards the glycolysis pathway [15].

However, the direct effects of SGLT2 inhibitor and HIF-PHI on podocytes are incompletely understood. Preliminary clinical observation suggests that the two classes of drugs may be additive or synergistic in their effects [18]. In this study, we investigated the effects of an SGLT2 inhibitor Dapagliflozin and HIF-PHI Roxadustat on the expression and distribution of podocyte-specific molecules and cytoskeleton in cultured human podocytes as well as kidney biopsies from human DKD.

### Patients and methods

This study was approved by the Joint Chinese University Hong Kong-New Territories East Cluster Clinical Research Ethics Committee (approval number CRE-2022.684). All study procedures were in compliance with the Declaration of Helsinki.

### Cell culture and treatments

Conditionally immortalized human podocytes were provided by an established source [19]. Cells were cultured under growth-permissive conditions at 33 °C in RPMI 1640 Medium (Invitrogen) supplemented with 10% Fetal Bovine Serum (FBS), 1× Insulin, Transferrin, and Sodium Selenite (ITS), and 1× Penicillin-Streptomycin (all from Invitrogen). To induce differentiation, podocytes were grown and maintained in nonpermissive conditions at 37 °C for 14 days. Podocytes between passages 1 and 3 were used in all experiments. To establish podocyte injury in vitro, the differentiated podocytes were starved in medium containing 1% FBS for 12 h first and then subjected to different stimuli for 48 h: (1) normal glucose (NG, 5 mM); (2) high glucose (HG, 25 mM); (3) Dapagliflozin (1.1 nM or 11 nM, MedChemExpress); (4) Roxadustat (3 µM or 30 µM, MedChemExpress). The choice of glucose concentrations was based on the usual physiological concentration in plasma and a previous studies [20, 21]. The concentrations of Dapagliflozin and Roxadustat were those reported in previous reports [22, 23].

### Human diabetic kidney disease

We recruited 10 patients with type 2 diabetes mellitus and biopsy-proven diabetic kidney disease; 5 of them received SGLT2i treatment for at least 3 months before kidney biopsy (treated group), while the other 5 did not have SGLT2i (untreated control group). Kidney biopsy was performed because of nephrotic range proteinuria in diabetic patients without retinopathy. Their demographic and clinical information were reviewed (refer to Table 1). The estimated glomerular filtration rate (eGFR) was calculated using the Chronic Kidney Disease Epidemiology Collaboration (CKD-EPI) equation [24]. The severity of glomerulosclerosis and tubulointerstitial fibrosis was assessed by standard morphometric analysis as described previously [25, 26].

**Table 1:** Demographic and baseline clinical data

Group	Control	SGLT2i	*P* value
No. of subjects	5	5	
Age (year)	65.4 ± 16.1	61.1 ± 14.2	0.688 ^a^
Proteinuria (g/day)	5.31 ± 3.32	5.90 ± 2.84	0.769 ^a^
Serum creatinine (µmol/l)	147.6 ± 84.9	153.6 ± 91.8	0.917 ^a^
Estimated GFR (ml/min/1.73 m^2^)	49.2 ± 28.9	51.3 ± 29.2	0.911 ^a^
HbA1c (%)	7.3 ± 0.6	7.1 ± 0.6	0.630 ^a^
Histological damage (%)			
Glomerulosclerosis	22.1 ± 19.1	30.5 ± 18.0	0.496 ^a^
Tubulointerstitial fibrosis	19.0 ± 7.4	22.0 ± 9.1	0.583 ^a^
Lipid profile (mmol/L)			
Total cholesterol	5.1 ± 1.3	5.1 ± 1.5	0.931 ^a^
LDL cholesterol	3.2 ± 1.2	3.4 ± 1.4	0.806 ^a^
HDL cholesterol	1.1 ± 0.2	1.2 ± 0.3	0.525 ^a^
Triglyceride	2.5 ± 1.7	1.3 ± 0.2	0.150 ^a^
Comorbidities, no of cases (%)			
Ischemic heart disease	2 (40.0)	2 (40.0)	1.000 ^b^
Peripheral vascular disease	1 (20.0)	0	0.292 ^b^
Congestive heart failure	2 (40.0)	2 (40.0)	1.000 ^b^
Cerebrovascular accident	1 (20.0)	0	0.292 ^b^
Peripheral neuropathy	2 (40.0)	0	0.114 ^b^
Retinopathy	4 (80.0)	2 (40.0)	0.197 ^b^
Treatment for diabetes, no of cases (%)			
DPP4 inhibitor	2 (40.0)	1 (20.0)	0.490 ^b^
GLP1ra	0	0	—
Sulfonylurea	3 (60.0)	4 (80.0)	0.490 ^b^
Thiazolidinedione	1 (20.0)	0	0.292 ^b^
Metformin	4 (80.0)	3 (60.0)	0.490 ^b^
Insulin	1 (20.0)	1 (20.0)	1.000 ^b^
Other medications, no. of case (%)			
Statin	2 (40.0)	2 (40.0)	1.000 ^b^
Aspirin	3 (60.0)	2 (40.0)	0.527 ^b^
RAAS blocker	5 (100.0)	5 (100.0)	—
Diuretics	5 (100.0)	1 (20.0)	0.010 ^b^

GFR, glomerular filtration rate; LDL, low density lipoprotein; HDL, high density lipoprotein; DPP4, dipeptidyl-peptidase-4; GLP1ra, glucagon-like peptide-1 receptor agonists; RAAS, renin-angiotensin-aldosterone system

Data were compared by ^a^unpaired Student’s t test and ^b^Chi-square test

### Immunofluorescent staining

Cultured podocytes and paraffin sections of human kidney specimen (4 μm thick) were fixed and stained with primary antibodies and followed by applying the Alexa Fluor™ 488 Tyramide SuperBoost™ Kit (Invitrogen) and Alexa Fluor™ 594 Tyramide SuperBoost™ Kit (Invitrogen). Primary antibodies include Nephrin (Invitrogen), Synaptopodin (Abcam, Cambridge, MA), Podocin (Novus Biologicals), Podocalyxin (Abcam), F-actin (Invitrogen), α-Actinine-4 (Novus Biologicals), and Wilms’s tumor-1 (WT-1, Santa Cruz Biotechnology). Sections were counter-stained with 4’,6-diamidino-2-phenylindole (DAPI) or Propidium Iodide (Thermo Scientific) and mounted with Prolong™ Glass Antifade Mountant (Invitrogen). Sections were examined using an LSM 880 Fluorescent Confocal Laser Microscope (Carl Zeiss AG). For dual-color staining, images were acquired sequentially to avoid dye interference. ImageJ Software (National Institutes of Health, USA) was used for postprocessing and the semi-quantitative analysis of the images. For kidney biopsy from two groups, at least 10 glomeruli/section for each patient were randomly acquired using confocal laser scanning microscopy, and the immunofluorescence staining intensities of each protein were performed by ImageJ software, the integrated pixel densities were used for comparison.

### Counting of glomerular podocyte number

Podocytes were identified as cells positive for WT-1. Kidney sections were incubated overnight with rabbit anti-WT-1 antibody (Santa Cruz Biotechnology), followed by Alexa Fluor™ 594 Tyramide SuperBoost™ Kit (Invitrogen). The estimation of the average number of podocytes per glomerulus was determined by morphometric analysis as previously described in our previous study [27]. At least 10 glomeruli/section for each patient were randomly acquired using confocal laser scanning microscopy.

### RNA extraction and RT-qPCR assay

Total RNA from the cultured podocytes was extracted by RNeasy mini kit (Qiagen Sciences). The cDNA was prepared by the SuperScript™ IV FirstStrand Synthesis System (ThermoFisher Scientific). Quantitation of mRNA was performed with the StepOnePlus real-time PCR system (Applied Biosystems) using TaqMan Fast Advanced Master Mix (ThermoFisher Scientific). Commercially available TaqMan primers and probes were used for all target genes (all from ThermoFisher Scientific). Each sample was run in triplicate. 18 S rRNA was used as the housekeeping gene. Results were analyzed with the use of Sequence Detection software, version 1.9 (Applied Biosystems). Gene expression for each signal was calculated by using the difference-in-threshold-cycle procedure. For the quantification of the target mRNA abundance, differences of threshold cycles between target and housekeeping gene were calculated by 2^−ΔΔCT^ method.

### ELISA

Cell free nephrin, podocin, podocalyxin and synaptopodin levels in the cell culture supernatant were measured by the human PDCN ELISA kit, human SYNPO ELISA kit, human PCX ELISA kit and human NPHN ELISA kit (all from Biorbyt). All assays were performed in duplicate according to the manufacturer’s instructions. The inter-assay coefficient of variation meets the requirements given in the manufacturer’s instructions.

### Statistical analysis

Results are expressed as mean ± SEM unless otherwise specified. Data analysis was performed with Prism Software (GraphPad Software Inc.). Comparisons between groups were made by one way analysis of variance (ANOVA) or Chi square test as appropriate. Post hoc comparison between groups was performed by Student’s t test with adjustment for multiple comparison by the Benjamini-Hochberg procedure. Statistical significance was defined as *P* < 0.05. All probabilities were two tailed.

## Results

### Pattern of distribution of podocyte-associated molecules

**Fig. 1:**
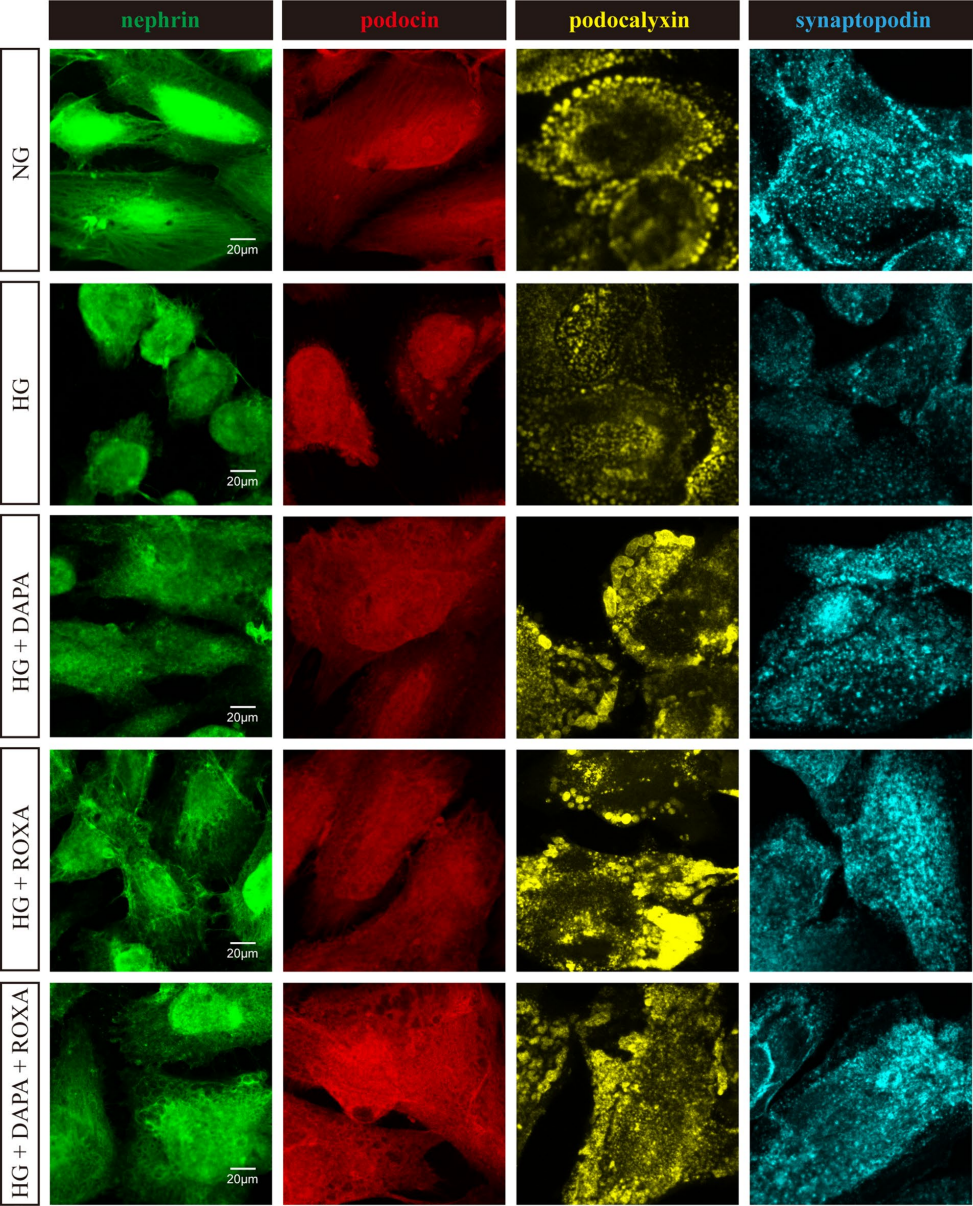
Effects of high glucose condition, Dapagliflozin, Roxadustat, and combined therapy on the distribution pattern of podocyte-specific molecules in cultured human podocytes. Representative images for nephrin, podocin, podocalyxin, and synaptopodin. Original magnification, ×630. Scale bar: 20 μm. NG, normal glucose (5 mM), HG, high glucose (25 mM), DAPA, Dapagliflozin (11 nM), ROXA, Roxadustat (30 µM)

The pattern of expression of podocyte-associated molecules in cultured podocytes was evaluated by indirect immunofluorescence staining (Fig. 1). Under normal glucose concentration, nephrin and podocin staining were strong at the cell periphery, appeared to be filamentous, and distributed equally all around the cell periphery. Under high glucose concentration, podocyte cell bodies were shrunken, and the expression of nephrin and podocin on the cell surface were substantially reduced and became granular in appearance. Dapagliflozin, Roxadustat, or their combination restored the expression and distribution of nephrin and podocin in podocytes, and the effects was dose-dependent (Figures S1 and S2).

Under normal glucose concentrations, podocalyxin expression was observed along the apical surface of the cell body and foot processes. This is coupled with the appearance of open intercellular spaces. When exposed to high glucose concentration, podocalyxin at apical cell membrane decreased and appeared disorganized (Fig. 1). Treatment with Dapagliflozin, but not Roxadustat, partly restored the patchy distribution of podocalyxin, and the effect was dose-dependent (Figure S3).

For synaptopodin, there was a strong expression in the cytoplasm, which extended into cell processes in a punctate pattern in normal glucose concentration. When exposed to high glucose concentration, the expression of synaptopodin was substantially reduced, especially at the cell processes, and the punctate appearance was disrupted (Fig. 1). Treatment with Dapagliflozin, and less with Roxadustat, the expression and distribution pattern of synaptopodin was restored in a dose-dependent manner (Figure S4).

**Fig. 2:**
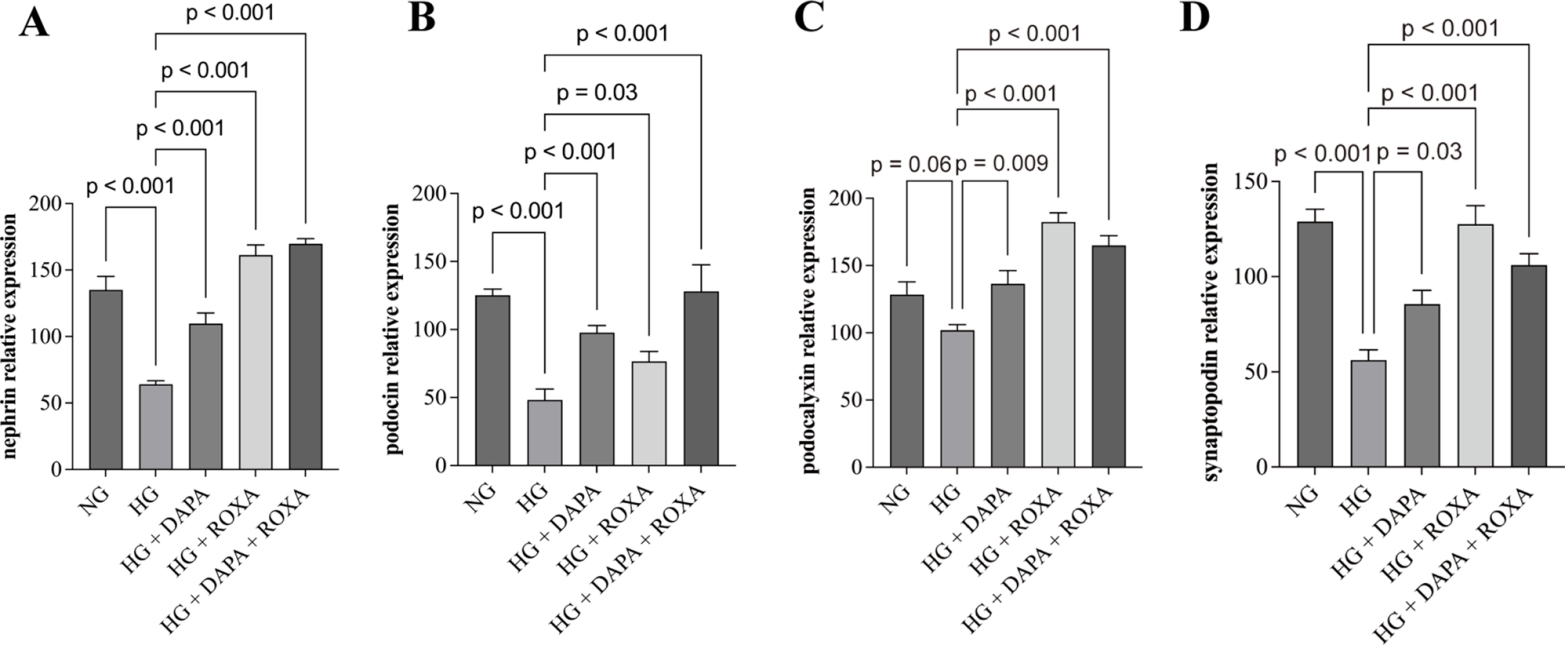
Intracellular protein levels of podocyte-specific molecules in cultured human podocytes as determined by indirect immunofluorescence staining: (**A**) nephrin; (**B**) podocin; (**C**) podocalyxin; and (**D**) synaptopodin. Quantification of glomerular nephrin, podocin, podocalyxin, and synaptopodin level by semiquantitative computerized image analysis. Error bars denote standard error of mean (SEM); data were compared by one way analysis of variance (ANOVA) (overall *p* < 0.01); post hoc comparison between groups was performed by unpaired Student’s t test with adjustment for multiple comparison by the Benjamini-Hochberg procedure. NG, normal glucose (5 mM), HG, high glucose (25 mM), DAPA, Dapagliflozin (11 nM), ROXA, Roxadustat (30 µM)

**Fig. 3:**
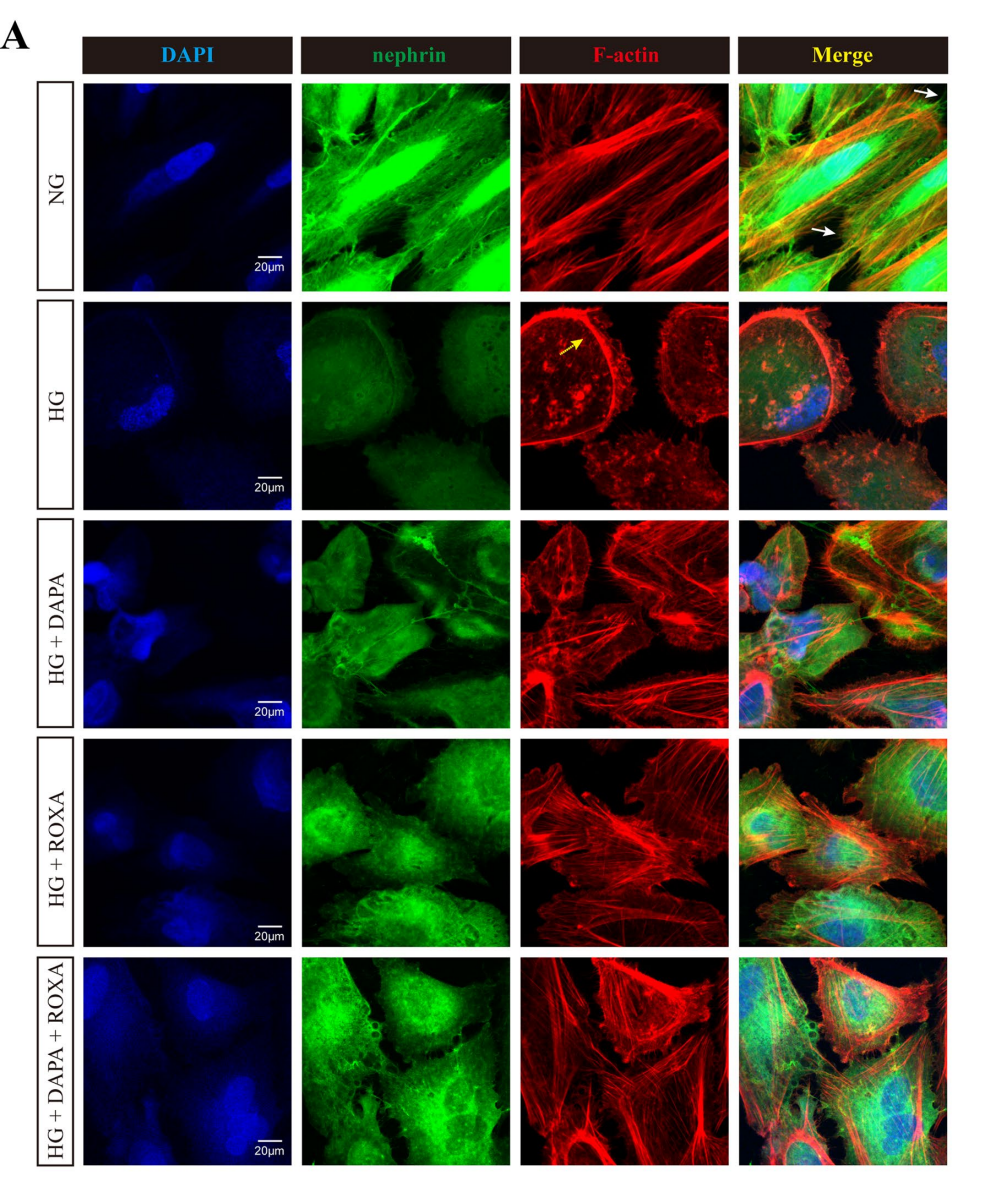

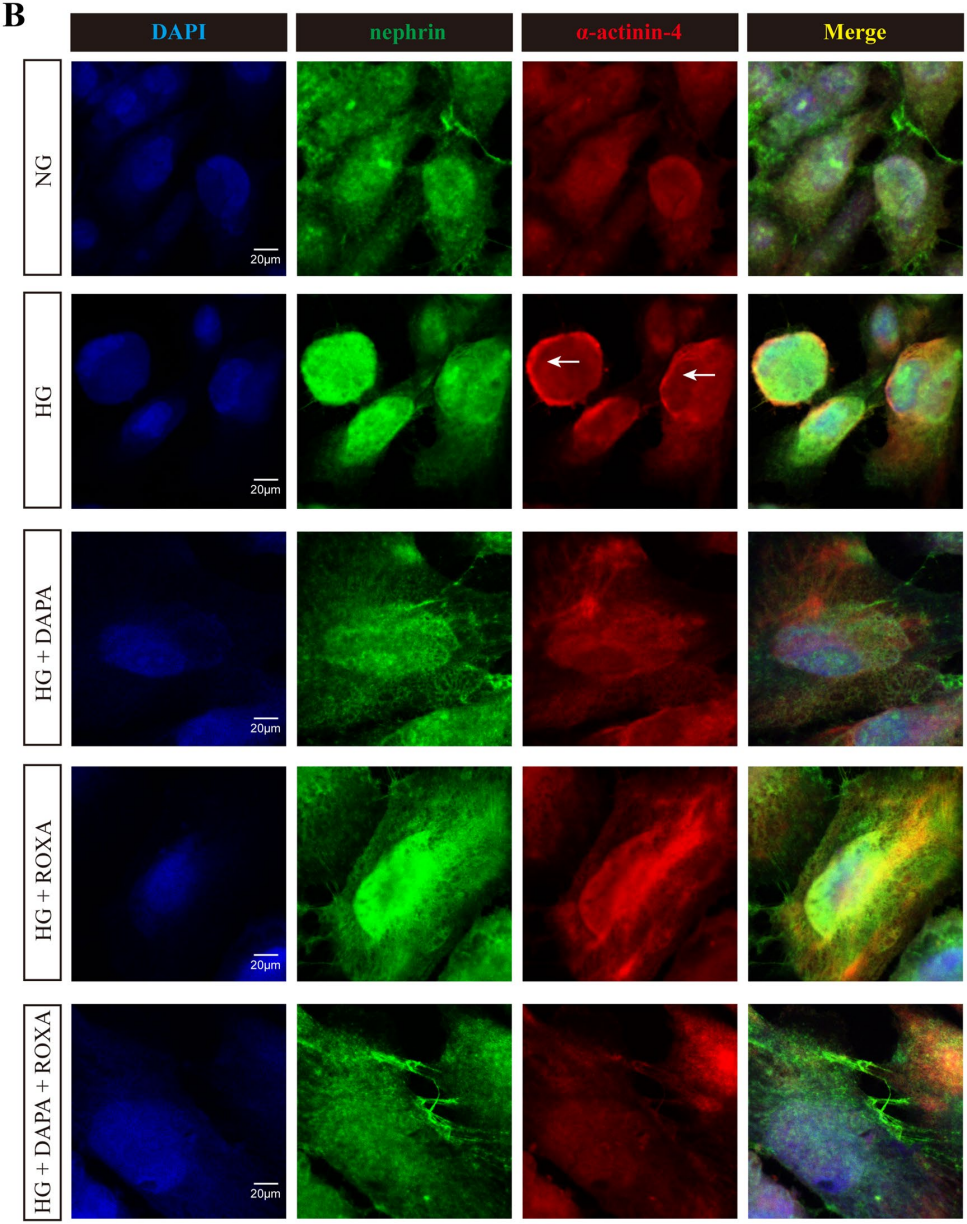

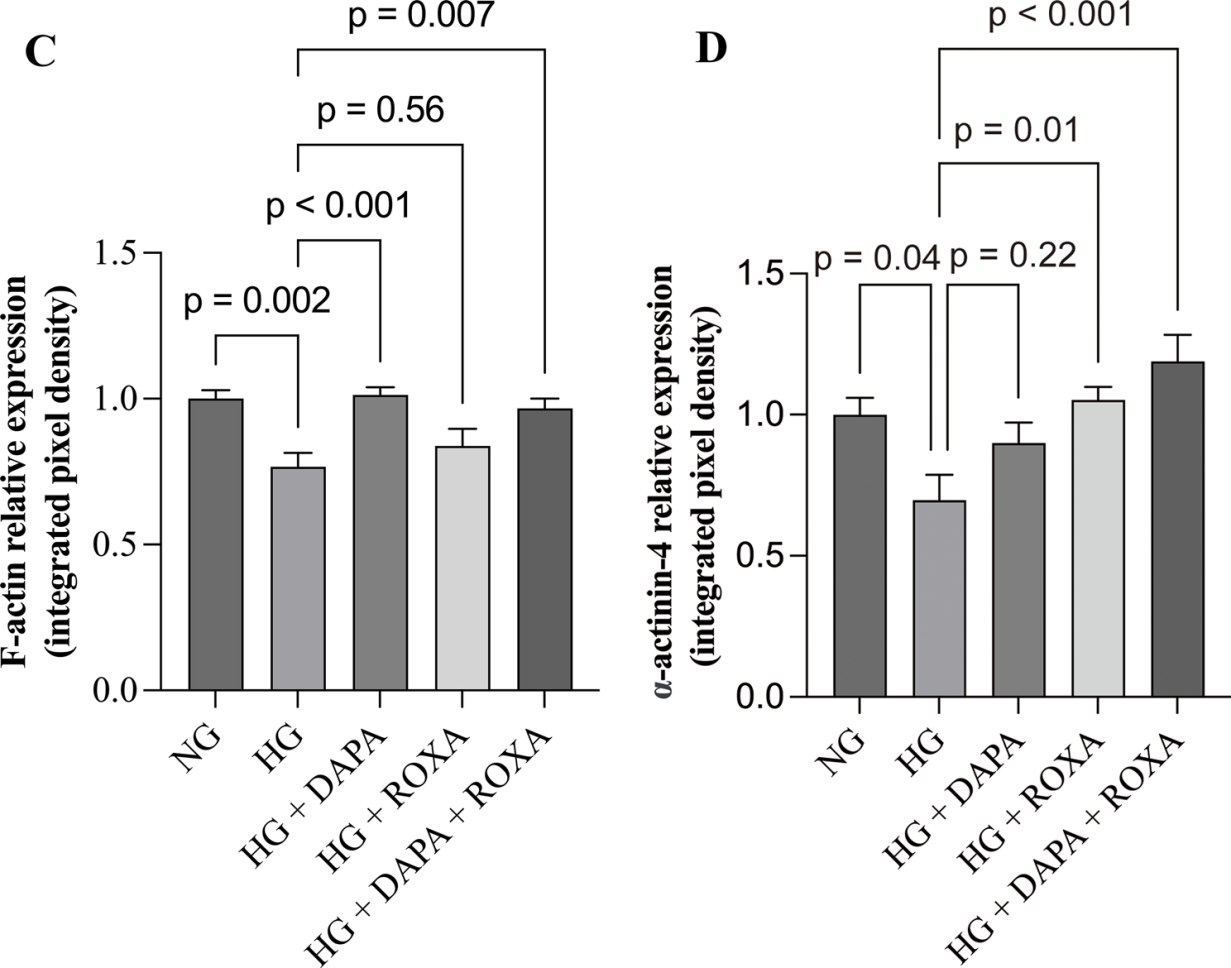
Effect of high glucose condition, Dapagliflozin, Roxadustat, and combined therapy on the distribution of F-actin and α-actinin-4 in cultured human podocytes: (**A**) representative images for nephrin (green) and F-actin (red); yellow arrow: podocytes with F-actin relocation; white arrow: co-localization of nephrin and F-actin; (**B**) representative images for nephrin (green) and α-actinin-4 (red); arrows: podocytes with α-actinin-4 relocation. Nuclei were counterstained with DAPI (blue). Original magnification, ×630. Scale bar: 20 μm. The immunofluorescence staining intensities and quantification of glomerular (**C**) F-actin and (**D**) α-actinin-4 levels were measured by semiquantitative computerized image analysis. The intensities of all other treatments are normalized to NG levels. Error bars denote standard error of mean (SEM); data were compared by one way analysis of variance (ANOVA) (overall *p* < 0.01); post hoc comparison between groups was performed by unpaired Student’s t test with adjustment for multiple comparison by the Benjamini-Hochberg procedure. NG, normal glucose (5 mM), HG, high glucose (25 mM), DAPA, Dapagliflozin (11 nM), ROXA, Roxadustat (30 µM)

**Fig. 4:**
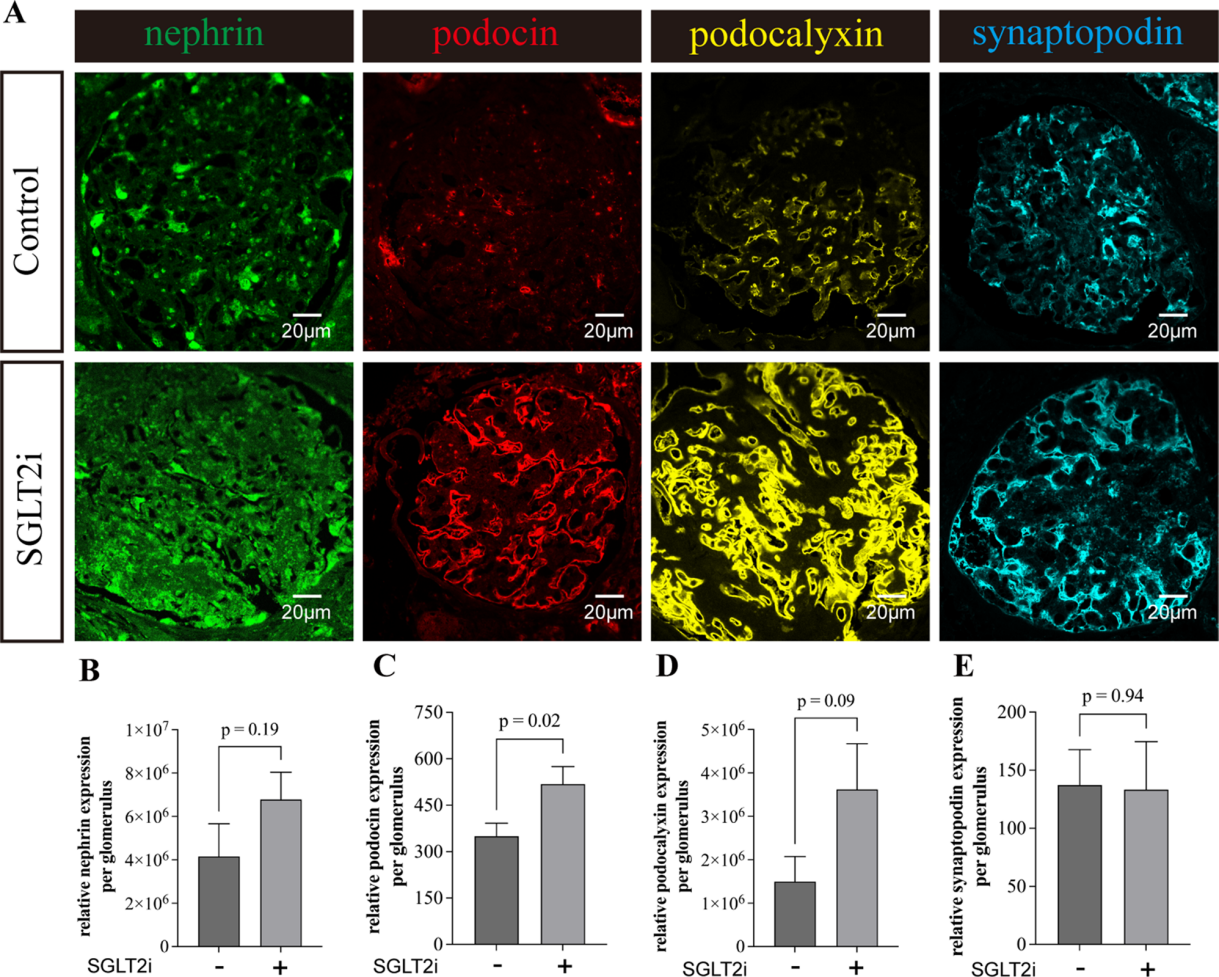
Glomerular distribution pattern of podocyte-specific molecules in in human kidney specimen from patients with diabetic kidney disease, with or without sodium-glucose co-transporter 2 inhibitor (SGLT2i) therapy (5 patients in each group). (**A**) Representative images for nephrin, podocin, podocalyxin, and synaptopodin. Quantification of glomerular (**B**) nephrin, (**C**) podocin, (**D**) podocalyxin, and (**E**) synaptopodin levels were measured by semiquantitative computerized image analysis. Original magnification, ×630. Scale bar: 20 μm. Error bars denote standard error of mean (SEM) of all 5 cases from each group; data were compared by one way analysis of variance (ANOVA) (overall *p* < 0.01); post hoc comparison between groups was performed by unpaired Student’s t test with adjustment for multiple comparison by the Benjamini-Hochberg procedure

### Quantity of podocyte-associated molecules

The intracellular mRNA levels of podocyte-associated molecules and their corresponding levels in culture supernatant were then evaluated. Cultured podocytes subjected to high glucose condition had reduced mRNA expression of podocyte-specific molecules (i.e. nephrin, podocalyxin and synaptopodin), while treatment with Dapagliflozin, Roxadustat, or their combination led to a significant increase (Figure S5). The effect of Dapagliflozin and Roxadustat on the mRNA expression of podocyte-specific molecules was dose-dependent (Figure S6). No results of podocin mRNA expression due to insufficient sample quantity. On the other hand, intracellular protein levels of podocyte-associated molecules as quantified by semiquantitative computerized image analysis were significantly reduced when podocytes were subjected to high glucose condition (although the change in podocalyxin level did not reach statistical significance), while Dapagliflozin, Roxadustat, or their combination restored their intracellular protein levels (Fig. 2). On the other hand, there was no significant change in the level of cell-free podocin or synaptopodin levels in the culture supernatant as measured by ELISA (Figure S7). Nephrin and podocalyxin were not detected in the culture supernatant.

### Distribution of cytoskeleton

The intracellular distribution of cytoskeleton in cultured podocytes was then examined by immunofluorescence. Under normal glucose concentration, F-actin filaments appeared as stress fiber-like bundles along the axis of the podocytes or into the process of arborized cells (Fig. 3). Co-localization of nephrin and F-actin was observed on podocyte surface or the nearby cytoplasm. Exposure to high glucose concentration led to reorganization of the F-actin, which became concentrated at the periphery of the podocytes, with the filamentous pattern disappeared. Treatment with Roxadustat, and less markedly with Dapagliflozin, restored the filamentous distribution of F-actin and its co-localization with nephrin in a dose-dependent manner (Figure S8).

For α-actinin-4, the distribution under normal glucose concentration was diffuse, slightly filamentous throughout the cell, and partially co-localized with nephrin (Fig. 3). Exposure to high glucose induced re-localization of α-actinin-4 to the periphery of cells. Treatment with Dapagliflozin, Roxadustat, or their combination treatments only had a modest effect on restoring the distribution of α-actinin-4 (Figure S9).

### SGLT2 inhibitor therapy on podocyte morphology in human glomeruli

Next, we studied the effect of SGLT2i therapy on the podocyte morphology by immunofluorescence study of the kidney biopsy specimens of 10 DKD patients; 5 of them received SGLT2i therapy (Dapagliflozin 10 mg daily in all 5 cases) for at least 3 months before the biopsy. The baseline demographic and clinical data of the patients are summarized in Table 1. In essence, there was no significant difference in renal tissue damage, kidney function, proteinuria, severity of histological damage, concomitant medications, or glycemic control between the SGLT2i treated and control groups. Podocytes number was also similar between the groups (Figure S10).

The distribution of podocyte-specific molecules are determined by immunofluorescent staining (Fig. 4). In the group without SGLT2i treatment, nephrin exhibits a granular, discontinuous, and segmental pattern of distribution rather than the extensively arborized pattern as expected in normal glomeruli. In the SGLT2i treated group, the pattern of nephrin staining assumed a linear and continuous distribution. Similarly, the intensity of podocin, podocalyxin, and synaptopodin staining were low and had a granular distribution in the untreated group (Fig. 4). In the SGLT2i treated group, their intensity of staining was substantially increased and exhibited a strong linear continuous pattern.

## Discussion

In this study, we observed that cultured podocytes exposed to high glucose condition demonstrated resulted in abnormal mRNA expressions and intracellular protein levels of nephrin, podocalyxin, and synaptopodin. These abnormalities were partially reverted when treated with Dapagliflozin, Roxadustat, or a combination of both. Under hyperglycemic conditions, these treatments also reinstated the distribution of nephrin and podocin within podocytes and reversed podocyte body shrinkage. Dapagliflozin, but not Roxadustat, partially restored the normal distribution of podocalyxin and synaptopodin, contributing to the maintenance of cytoskeletal architecture in podocytes. Both Dapagliflozin and Roxadustat dose-dependently restore the expression and distribution of key podocyte markers—nephrin, podocin, and synaptopodin—disrupted by high glucose conditions. Dapagliflozin also partially restores podocalyxin distribution, while Roxadustat more effectively improves cytoskeletal organization, particularly F-actin structure. Their combined treatment shows additive benefits in reversing high glucose-induced podocyte injury. In human DKD, kidney biopsies also revealed disrupted distributions of nephrin, podocin, podocalyxin, and synaptopodin, while in DKD patients treated with SGLT2 inhibitors, the immunofluorescence staining pattern returned to a linear and continuous distribution.

DKD affects approximately 30–50% of patients with type 2 diabetes [3]. Renin-angiotensin system (RAS) blockers, including angiotensin-converting enzyme inhibitors (ACEIs) and angiotensin II receptor blockers (ARBs), have been pivotal in the management of proteinuria in DKD and non-diabetic CKD [28, 29]. In the past decade, SGLT2 inhibitors have emerged as another essential component of DKD treatment [30]. Clinical trials with kidney-related endpoints have shown that SGLT2 inhibitors reduce the composite indicators of renal failure and the increase of serum creatinine by 28% to 39% [30]. Notably, the renal benefits of these drugs occur independently of their glucose-lowering effects. For patients with advanced CKD, SGLT2 inhibitors have minimal glucose-lowering effect, yet still provide comparable long-term benefits [31, 32]. Evidence indicates that SGLT2 inhibition can reverse multiple DKD-related disorders and slow DKD progression [7–9]. These benefits include mitigating albuminuria, mesangial dilation, matrix accumulation, and interstitial fibrosis through combined effects on glomerular hemodynamics and the inhibition of renal inflammation and oxidative stress in experimental models of diabetes [33, 34].

On the other hand, the onset of diabetes has a direct impact on oxygen delivery to the kidneys [35]. Diabetes alters renal oxygen supply, impairing physiological adaptations including the activation of HIF-1α and metabolic reprogramming necessary for maintaining adenosine triphosphate (ATP) production [17]. Research by Hasegawa et al. has shown that the instability of HIF-1α and the disruption of its downstream compensatory pathway occur early in the progression of diabetic kidney disease [15]. Their study further illustrates that in diabetic kidneys, metabolic and transcriptional alterations were mitigated through the application of the HIF-1α stabilizer, enarodustat [15].

The integrity of podocytes is vital for maintaining a normal glomerular filtration barrier, and podocyte injury is a hallmark of DKD [36]. While specific research examining whether SGLT2 and HIF-PH inhibitors can restore podocyte abnormalities in diabetes is limited, our study provides insights to further support this notion. Our data suggests that podocytes are a favorably influenced by SGLT2 inhibitors and/or HIF-PH inhibitors. Notably, our findings align with previous reports [11]. Furthermore, we demonstrated that the combined therapy of these two drugs have additive effects in normalizing morphology and maintaining cytoskeleton architecture in podocytes.

In addition to its direct effects on the proximal renal tubules, there is cumulating evidence that SGLT2i preserves podocyte structural integrity, mitigates slit diaphragm dysfunction, decreases apoptosis, enhances podocyte autophagy, and thereby prevents podocyte loss in diabetic and non-diabetic CKD. Furthermore, SGLT2i may also attenuate lipotoxicity, oxidative stress, and inflammation in the glomerulus [37, 38]. In models of infectious kidney injury and oxidative stress-induced damage by decreasing NLRP3 inflammasome activity, SGLT2i exhibits anti-inflammatory properties [39, 40]. In a mouse model of type 2 diabetes, empagliflozin has been shown to augment the volume density of autophagosomes and autolysosomes in podocytes [41]. Dapagliflozin enhances AMPK activity while inhibits the mTOR pathway, thereby bolstering podocyte autophagy in diabetic nephropathy [42]. In this study, Dapagliflozin reduces foot process width and glomerular basement membrane thickness [42]. Similarly, empagliflozin has been found to increase podocyte number and density, decrease foot process width, and mitigate foot process effacement in diabetic nephropathy animal models [43]. In non-diabetic CKD, Dapagliflozin also limits podocyte injury by directly targeting podocytes, presumably via the maintenance of actin cytoskeleton structure [11]. In another study on a mouse model of western diet-induced obesity, Dapagliflozin reduces renal lipid accumulation and decreases podocyte damage markers [44]. The primary mechanism by which SGLT2i ameliorates lipotoxicity in podocyte involves augmenting free fatty acid utilization and reducing lipid accumulation, thereby preventing adipocyte detachment and apoptosis instigated by lipotoxicity [44]. Nonetheless, the mechanism of podocyte protection by SGLT2 inhibitor needs to be further tested, probably in diabetic mouse model.

The mechanism of podocyte retention by HIF-PHI also deserves further investigations. The podocyte retention mechanism by vascular endothelial growth factor (VEGF) is well reported [23]. Since HIF-PHI increases the transcriptional activity of VEGF, and VEGF is expressed in cultured podocyte [45] as well as mouse model of podocyte injury [46], there is a possibility of podocyte retention mediated by VEGF [47]. Recent research also indicates that the activation of HIF-1α can mitigate renal podocyte damage and reduce proteinuria in rats subjected to simulated high-altitude conditions [48]. Treatment with HIF-PHI in a simulated high-altitude environment led to a reduction in total urinary protein levels, probably via the facilitation of self-repair mechanisms by upregulating HIF-1α expression in podocytes [48]. In addition, following PHI administration, the increased expression of HIF-1α in renal tubules and interstitial tissues may promote podocyte repair through paracrine signalling.

Our study has several limitations. Firstly, the level podocyte-specific molecule in culture supernatant was measured as an indicator of sublethal podocyte injury, but the role of this marker has not been validated. Secondly, the human biopsy study was conducted as a single-center observational study with a limited sample size. Due to recruitment challenges, we do not have morphological data for podocytes from both a healthy control group and DKD patients undergoing treatment with HIF-PH inhibitors. We also did not quantify the number of glomerular podocytes before and after SGLT2i treatment in the podocyte culture experiments because the mechanism of podocyte death is very different between cell culture model and the in vivo situation. Because of the limited size of specimen for each patient, we were unable to quantify the mRNA expression levels of podocyte-associated molecules in human DKD specimens. Similarly, we did not quantify the mRNA expression level of inflammation, tissue injury, or oxidative stress markers because of the limitations in our original study design, and it would be interesting to compare these markers in Dapagliflozin and Roxadustat treated podocytes with the untreated ones. Furthermore, the range of kidney function of the DKD patients in this study was limited. Further research is essential to assess the effect of SGLT2i on podocytes across different stages of CKD. Similarly, additional studies are needed to evaluate whether HIF-PHI has a comparable effect on podocyte markers in patients with DKD and CKD.

In conclusion, our study showed that SGLT2 inhibitor Dapagliflozin and HIF-PH inhibitor Roxadustat are protective to podocytes in vitro, but the concomitant use of these two medications does not appear to have obvious additional effects. This treatment strategy may alleviate podocyte dysfunction and maintains podocyte cytoskeletal architecture in a diabetic milieu.

## Supplementary Information

Below is the link to the electronic supplementary material.

Supplementary Material 1

Supplementary Material 2

Supplementary Material 3

Supplementary Material 4

Supplementary Material 5

Supplementary Material 6

Supplementary Material 7

Supplementary Material 8

Supplementary Material 9

Supplementary Material 10

Supplementary Material 11

## Data Availability

All data generated or analyzed during this study are included in this article. Further enquiries can be directed to the corresponding author.
