# Supplementary figures and images for: The effect of SGLT2 inhibitor and HIF-PHI on the podocyte-specific molecules and cytoskeleton of diabetic podocytes
Source: BMC Nephrol. 2025 Dec 6;27:31. doi: 10.1186/s12882-025-04677-0 (PMC12797561; doi:10.1186/s12882-025-04677-0)

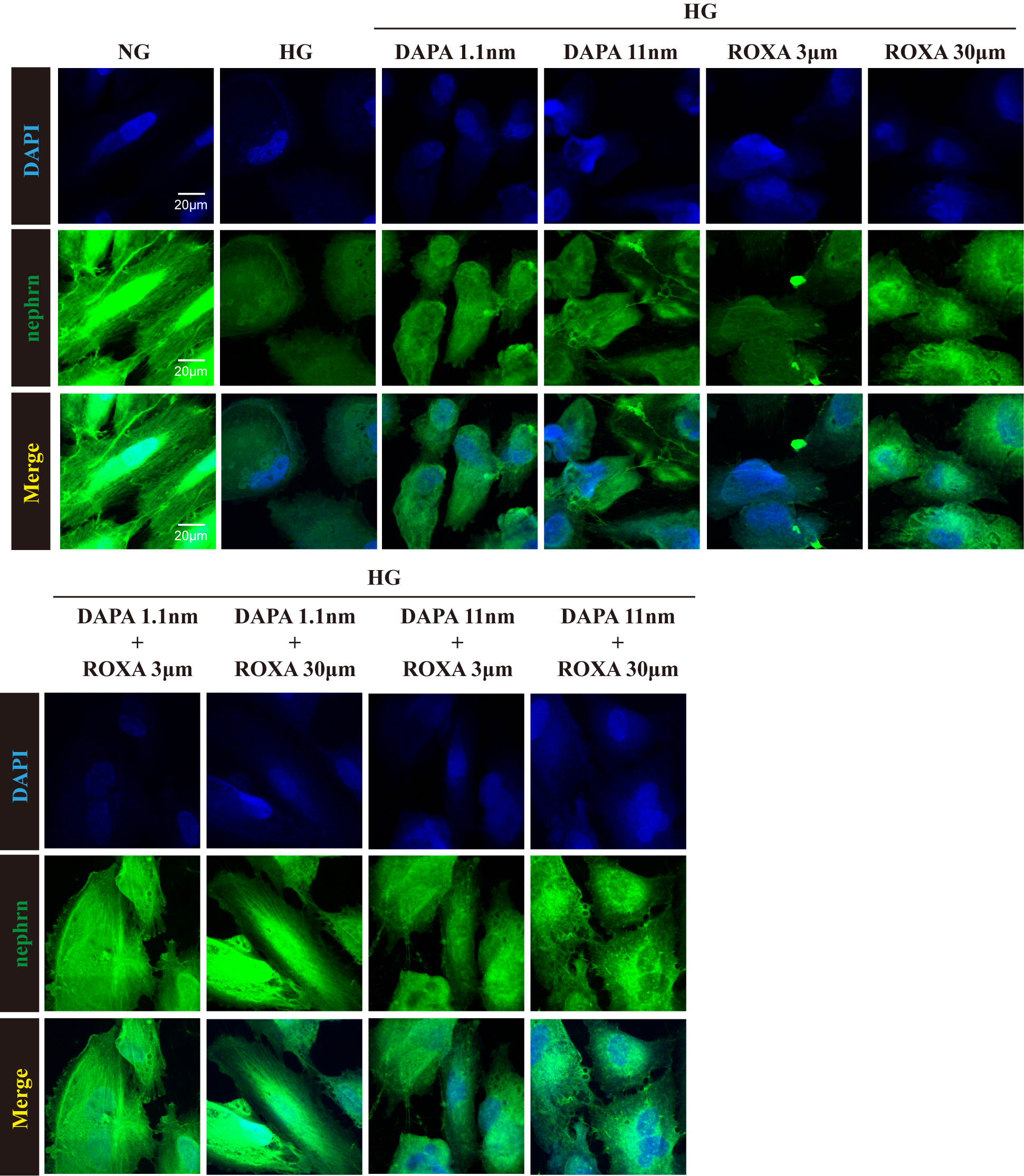

Supplement: Supplementary file 1 — Supplementary Material 1 [file 12882_2025_4677_MOESM1_ESM.tif]

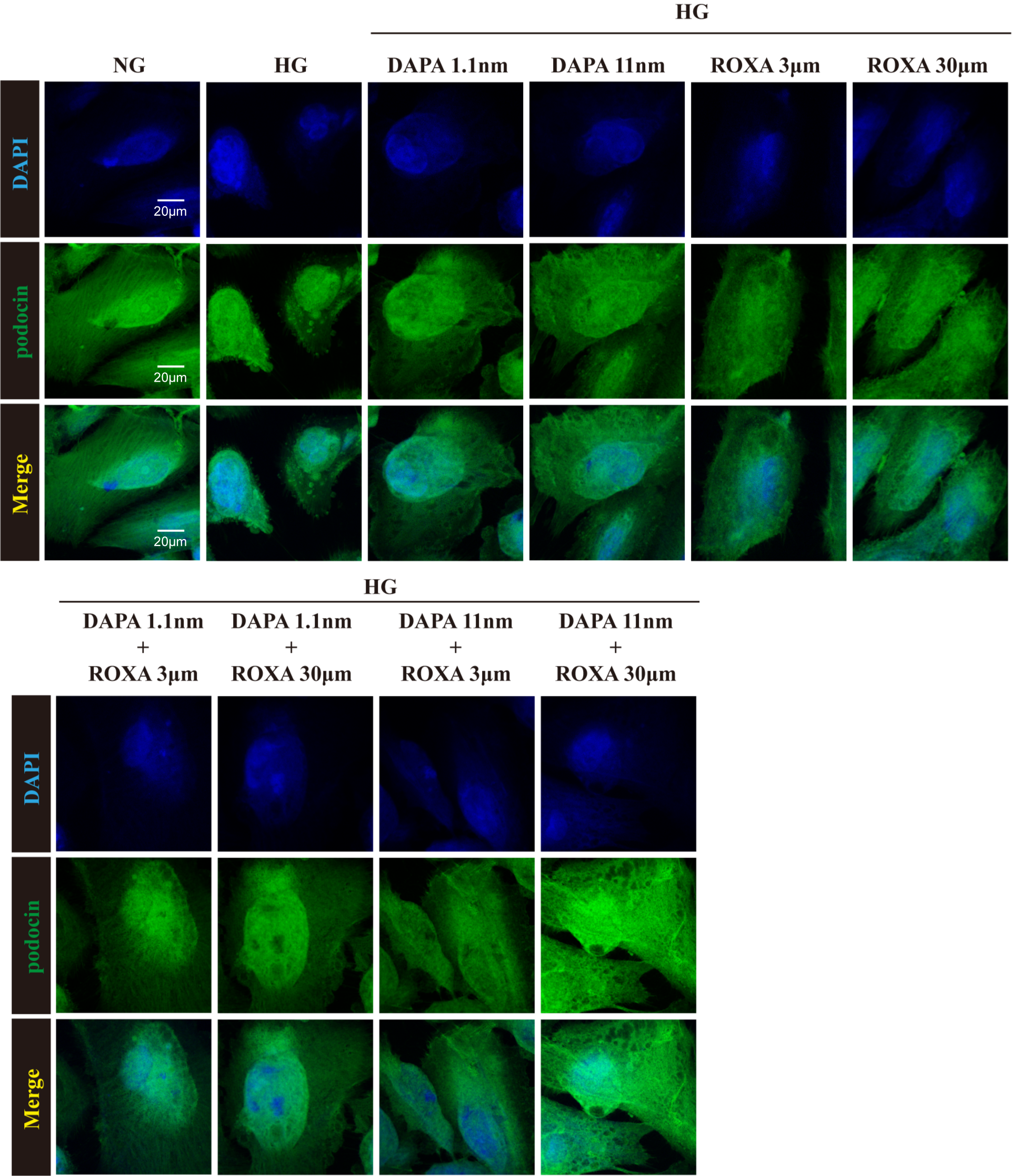

Supplement: Supplementary file 2 — Supplementary Material 2 [file 12882_2025_4677_MOESM2_ESM.tif]

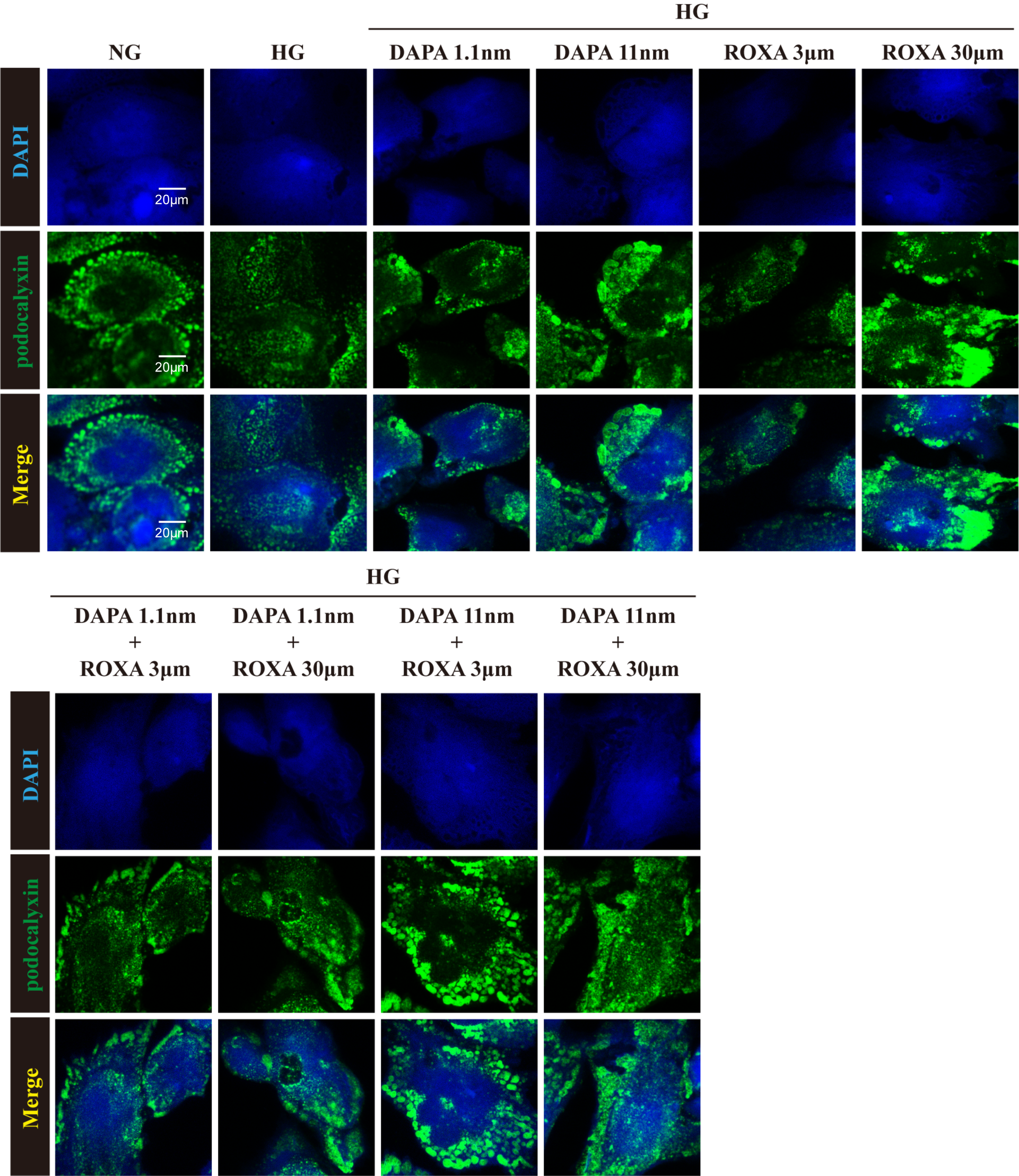

Supplement: Supplementary file 3 — Supplementary Material 3 [file 12882_2025_4677_MOESM3_ESM.tif]

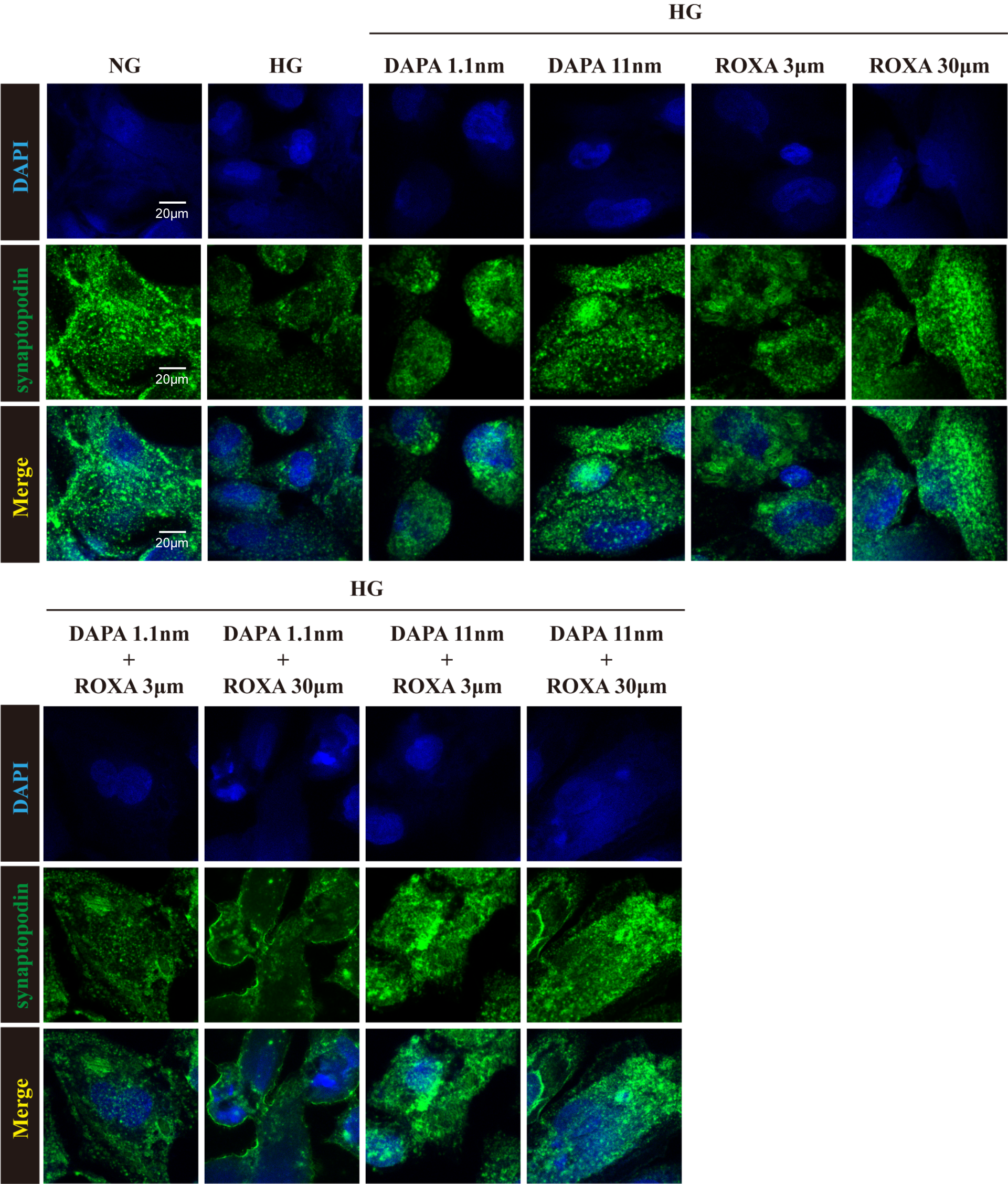

Supplement: Supplementary file 4 — Supplementary Material 4 [file 12882_2025_4677_MOESM4_ESM.tif]

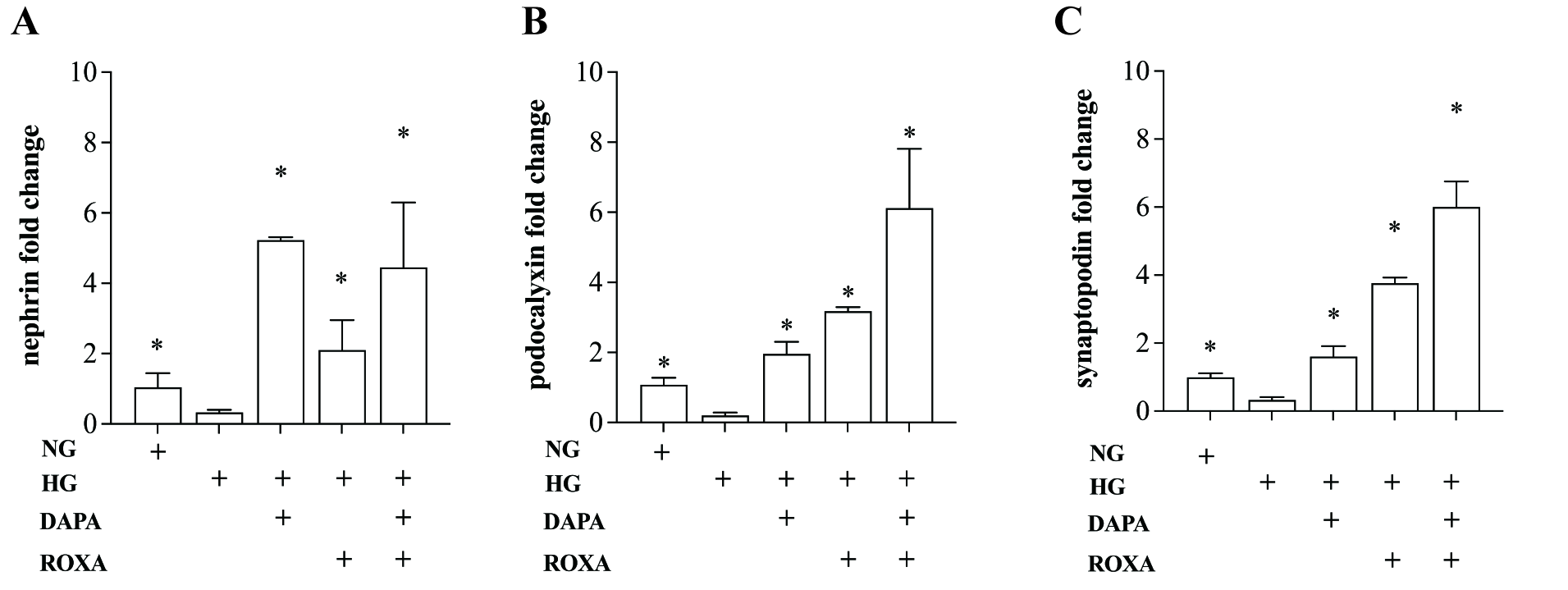

Supplement: Supplementary file 5 — Supplementary Material 5 [file 12882_2025_4677_MOESM5_ESM.tif]

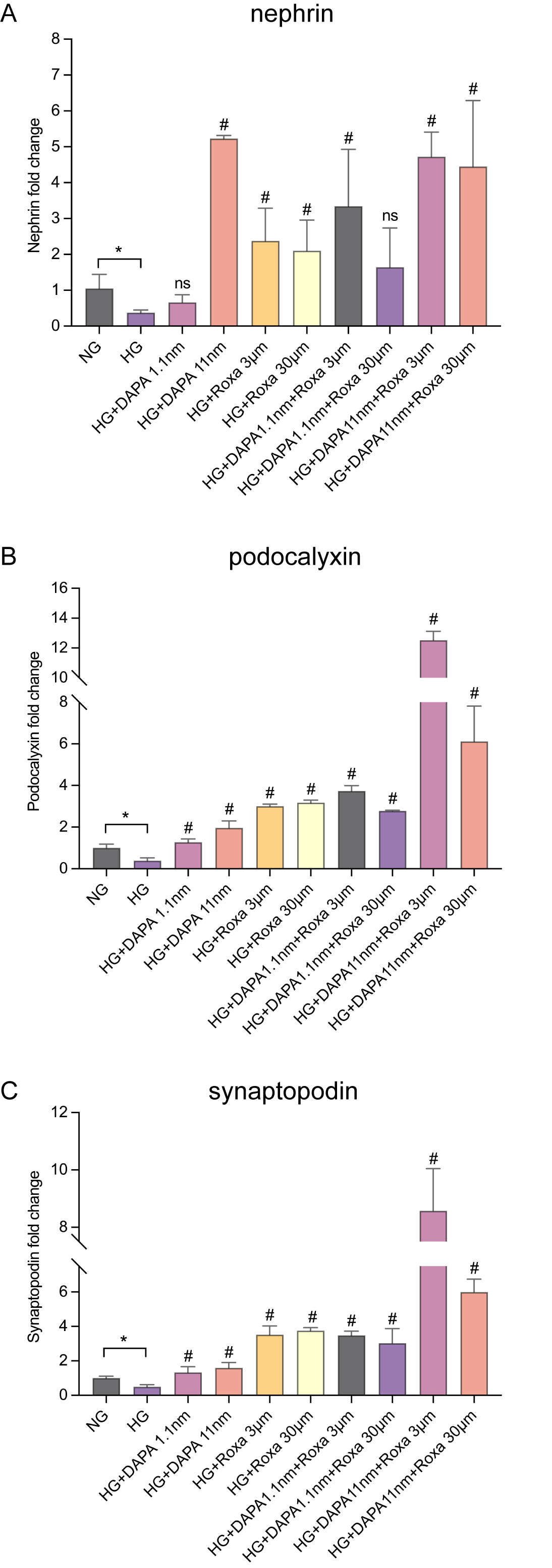

Supplement: Supplementary file 6 — Supplementary Material 6 [file 12882_2025_4677_MOESM6_ESM.tif]

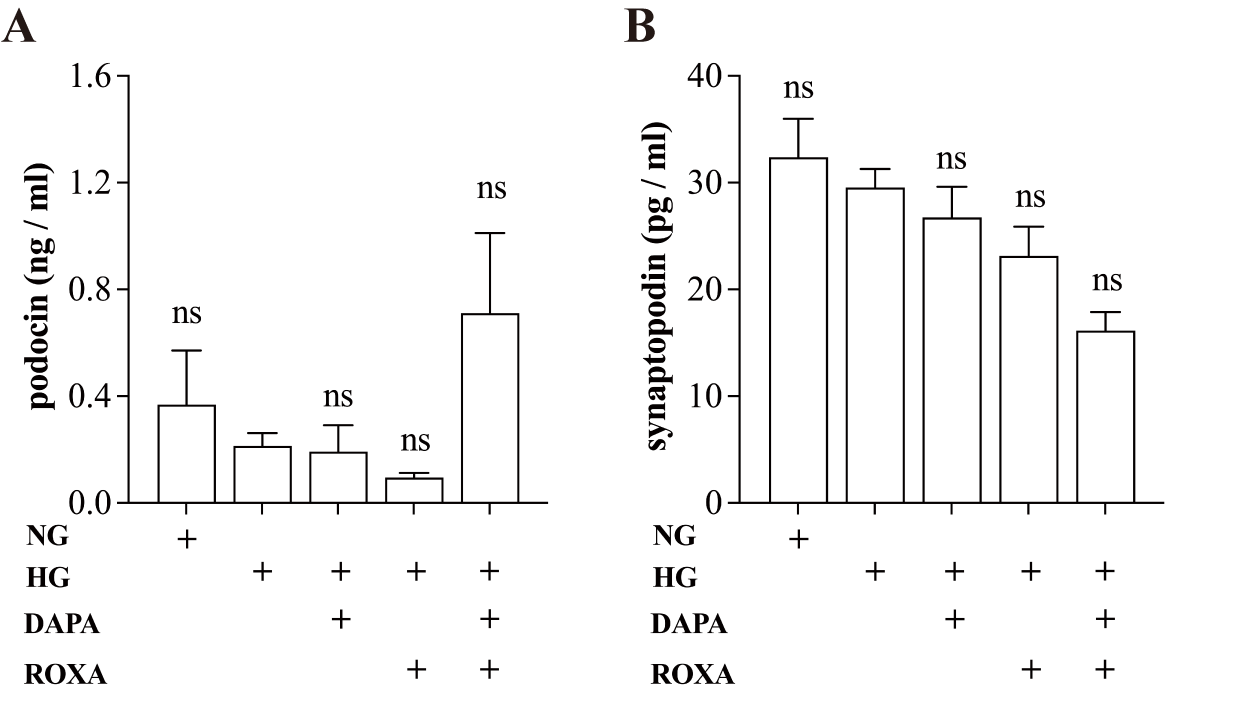

Supplement: Supplementary file 7 — Supplementary Material 7 [file 12882_2025_4677_MOESM7_ESM.tif]

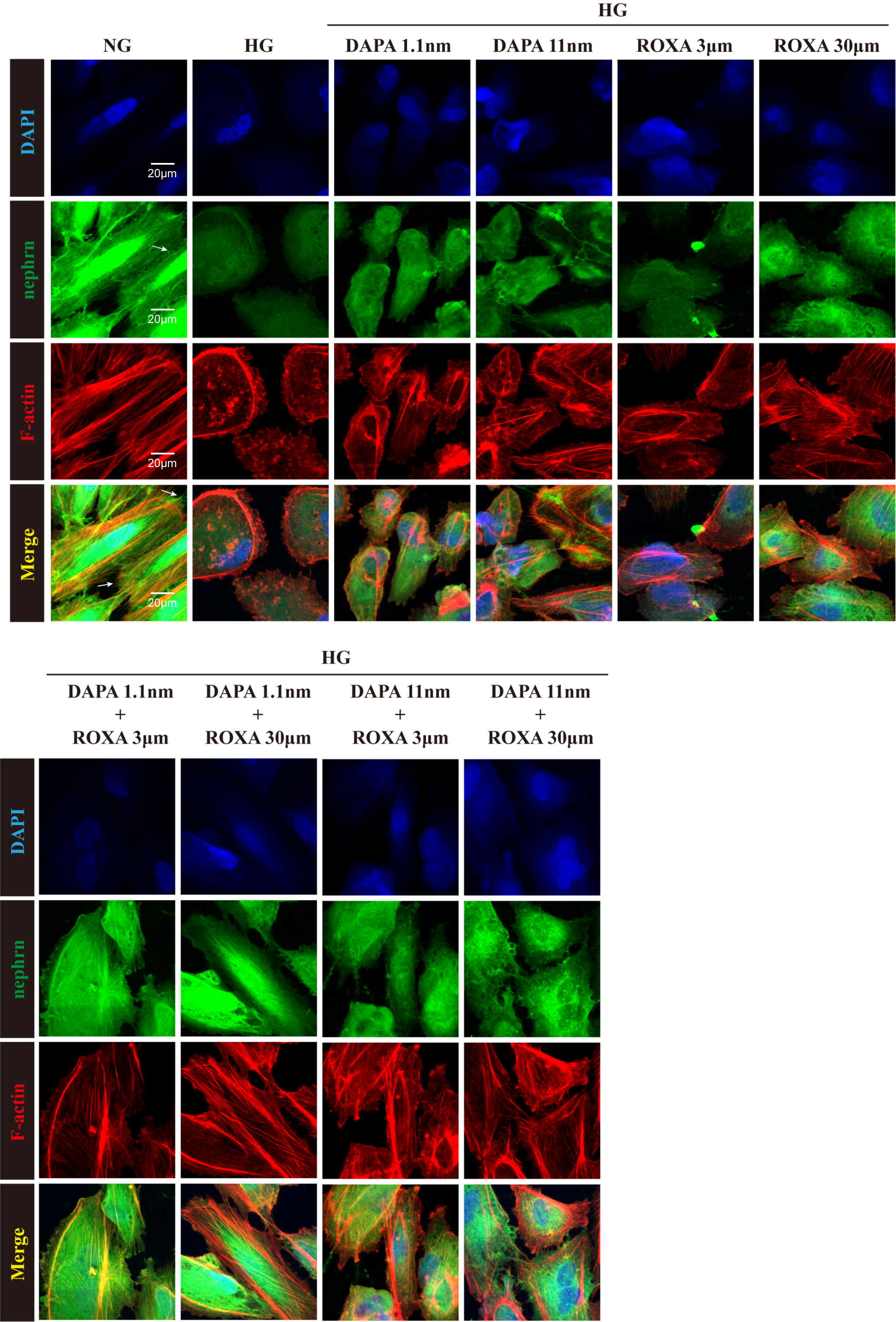

Supplement: Supplementary file 8 — Supplementary Material 8 [file 12882_2025_4677_MOESM8_ESM.tif]

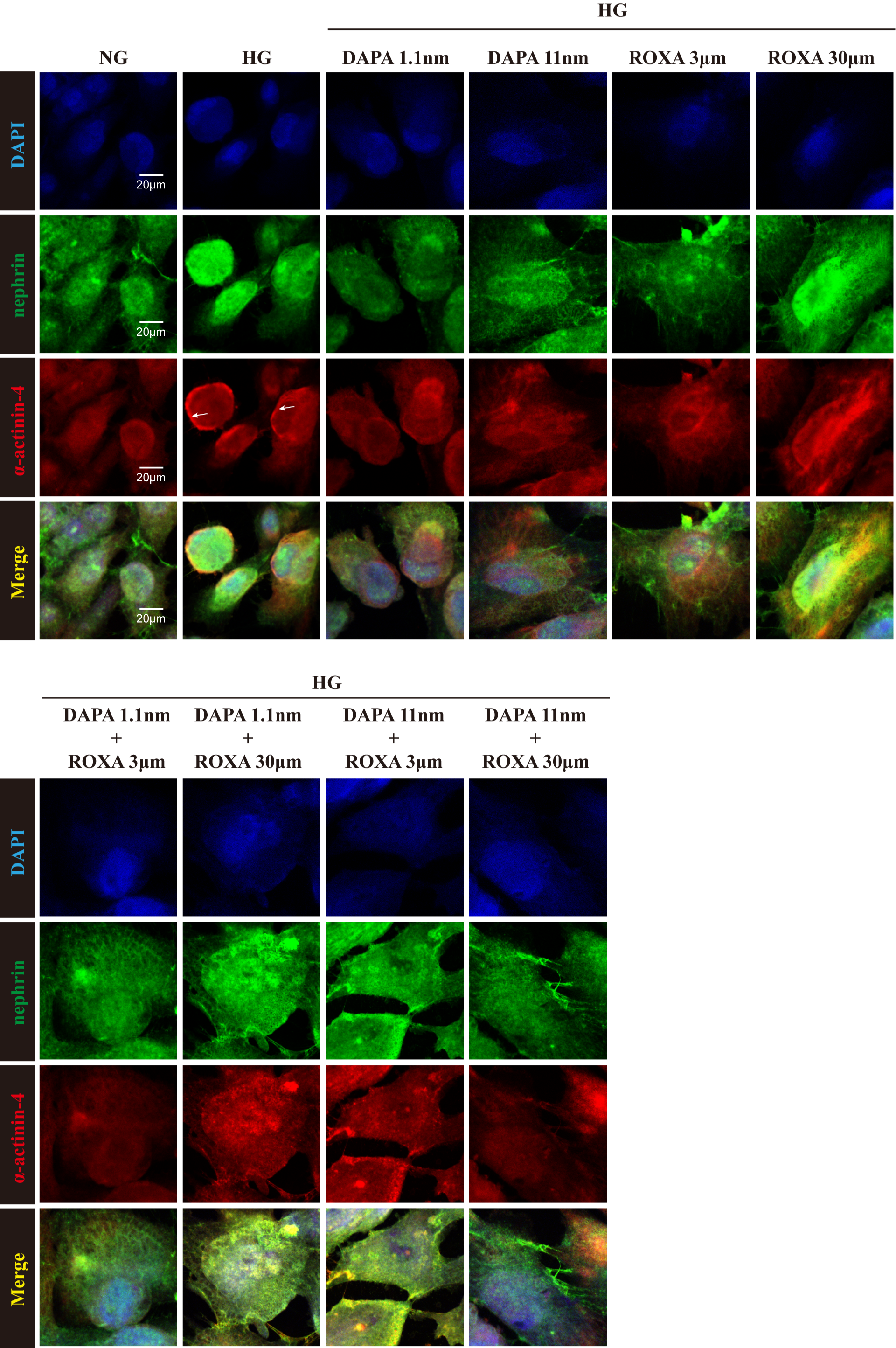

Supplement: Supplementary file 9 — Supplementary Material 9 [file 12882_2025_4677_MOESM9_ESM.tif]

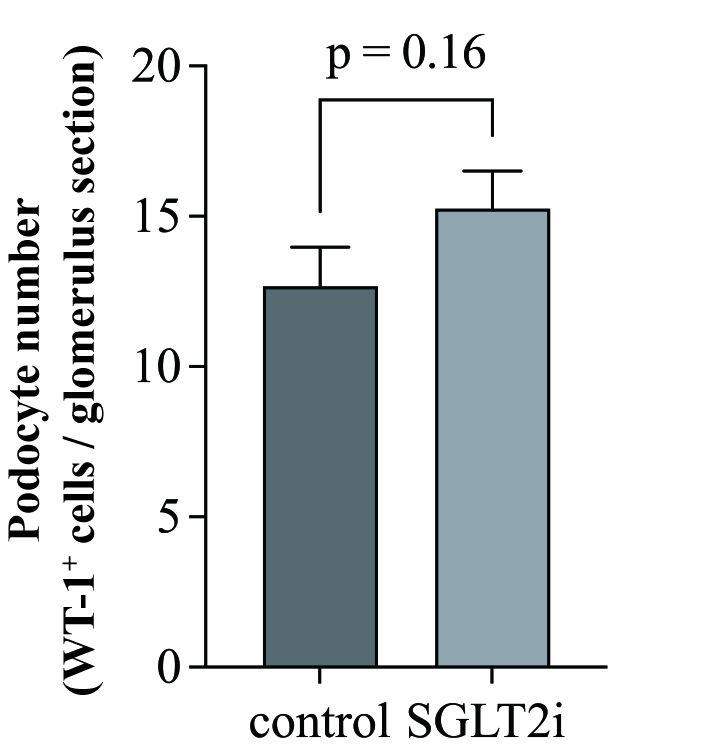

Supplement: Supplementary file 10 — Supplementary Material 10 [file 12882_2025_4677_MOESM10_ESM.tif]
