# Supplementary material for: The effect of SGLT2 inhibitor and HIF-PHI on the podocyte-specific molecules and cytoskeleton of diabetic podocytes
Source: BMC Nephrol. 2025 Dec 6;27:31. doi: 10.1186/s12882-025-04677-0 (PMC12797561; doi:10.1186/s12882-025-04677-0)
Supplement: Supplementary file 11 — Supplementary Material 11 [file 12882_2025_4677_MOESM11_ESM.docx]

**Supplementary materials**

**Figure S1:** Effect of high glucose condition, Dapagliflozin, Roxadustat, and combined therapy on the distribution pattern of nephrin in cultured human podocytes. Representative images for nephrin (green); Nuclei were counterstained with DAPI (blue). Original magnification, ×630. Scale bar: 20μm. NG, normal glucose (5 mM), HG, high glucose (25 mM), DAPA, Dapagliflozin (11 nM), ROXA, Roxadustat (30 μM).

**Figure S2:** Effect of high glucose condition, Dapagliflozin, Roxadustat, and combined therapy on the distribution pattern of podocin in cultured human podocytes. Representative images for podocin (green); Nuclei were counterstained with DAPI (blue). Original magnification, ×630. Scale bar: 20μm. NG, normal glucose (5 mM), HG, high glucose (25 mM), DAPA, Dapagliflozin (11 nM), ROXA, Roxadustat (30 μM).

**Figure S3:** Effect of high glucose condition, Dapagliflozin, Roxadustat, and combined therapy on the distribution pattern of podocalyxin in cultured human podocytes. Representative images for podocin (green); Nuclei were counterstained with DAPI (blue). Original magnification, ×630. Scale bar: 20μm. NG, normal glucose (5 mM), HG, high glucose (25 mM), DAPA, Dapagliflozin (11 nM), ROXA, Roxadustat (30 μM).

**Figure S4:** Effect of high glucose condition, Dapagliflozin, Roxadustat, and combined therapy on the distribution pattern of synaptopodin in cultured human podocytes. Representative images for podocin (green); Nuclei were counterstained with DAPI (blue). Original magnification, ×630. Scale bar: 20μm. NG, normal glucose (5 mM), HG, high glucose (25 mM), DAPA, Dapagliflozin (11 nM), ROXA, Roxadustat (30 μM).

**Figure S5:** The mRNA expression levels of podocyte-specific molecules in cultured human podocytes: (A) nephrin; (B) podocalyxin; and (C) synaptopodin. Data were presented as fold-change in gene expression relative to housekeeping gene (18s rRNA). Error bars denote standard error of mean (SEM); data were compared by unpaired Student’s t test. NG, normal glucose (5 mM), HG, high glucose (25 mM), DAPA, Dapagliflozin (11 nM), ROXA, Roxadustat (30 μM). *P < 0.05 as compared to the HG group.

**Figure S6:** The mRNA expression levels of podocyte-specific molecules in cultured human podocytes with different dosage of Dapagliflozin or Roxadustat: (A) nephrin; (B) podocalyxin; and (C) synaptopodin fold-change in gene expression relative to housekeeping gene. Error bars denote standard error of mean (SEM); data were compared by one way analysis of variance (ANOVA) (overall p < 0.01); post hoc comparison between groups was performed by unpaired Student’s t test with adjustment for multiple comparison by the Benjamini-Hochberg procedure. NG, normal glucose (5 mM), HG, high glucose (25 mM), DAPA, Dapagliflozin, Roxa, Roxadustat. *P < 0.05 as compared to NG group; # P < 0.05 as compared to the HG group; ns, difference not statistically significant.

**Figure S7:** Cell free level of (A) podocin; and (B) synaptopodin levels detected by ELISA in cell culture supernatant. Error bars denote standard error of mean (SEM); data were compared by one way analysis of variance (ANOVA) (overall p < 0.01); post hoc comparison between groups was performed by unpaired Student’s t test with adjustment for multiple comparison by the Benjamini-Hochberg procedure. NG, normal glucose (5 mM), HG, high glucose (25 mM), DAPA, Dapagliflozin (11 nM), ROXA, Roxadustat (30 μM); ns, difference not statistically significant.

**Figure S8:** Effect of high glucose condition, Dapagliflozin, Roxadustat, and combined therapy on the distribution pattern of nephrin and F-actin in cultured human podocytes. Representative images for nephrin (green) and F-actin (red), nuclei were counterstained with DAPI (blue). Original magnification, ×630. Scale bar: 20μm. NG, normal glucose (5 mM), HG, high glucose (25 mM), DAPA, Dapagliflozin (11 nM), ROXA, Roxadustat (30 μM).

**Figure S9:** Effect of high glucose condition, Dapagliflozin, Roxadustat, and combined therapy on the distribution pattern of nephrin and α-actinin-4 in cultured human podocytes. Representative images for nephrin (green) and α-actinin-4 (red), nuclei were counterstained with DAPI (blue). Original magnification, ×630. Scale bar: 20μm. NG, normal glucose (5 mM), HG, high glucose (25 mM), DAPA, Dapagliflozin (11 nM), ROXA, Roxadustat (30 μM).

**Figure S10:** Podocyte density in human kidney specimen from patients with diabetic kidney disease, with or without sodium-glucose co-transporter 2 inhibitor (SGLT2i) therapy (5 patients in each group). Error bars denote standard error of mean (SEM); data were compared by one way analysis of variance (ANOVA) (overall p < 0.01); post hoc comparison between groups was performed by unpaired Student’s t test with adjustment for multiple comparison by the Benjamini-Hochberg procedure.
